# miRNA-720 Controls Stem Cell Phenotype, Proliferation and Differentiation of Human Dental Pulp Cells

**DOI:** 10.1371/journal.pone.0083545

**Published:** 2013-12-30

**Authors:** Emilio Satoshi Hara, Mitsuaki Ono, Takanori Eguchi, Satoshi Kubota, Hai Thanh Pham, Wataru Sonoyama, Shoji Tajima, Masaharu Takigawa, Stuart K. Calderwood, Takuo Kuboki

**Affiliations:** 1 Department of Oral Rehabilitation and Regenerative Medicine, Okayama University Graduate School of Medicine, Dentistry and Pharmaceutical Sciences, Okayama-shi, Okayama-ken, Japan; 2 Department of Radiation Oncology, Division of Molecular and Cellular Biology, Beth Israel Deaconess Medical Center, Harvard Medical School, Boston, Massachusetts, United States of America; 3 Department of Biochemistry and Molecular Dentistry, Okayama University Graduate School of Medicine, Dentistry and Pharmaceutical Sciences, Okayama-shi, Okayama-ken, Japan; 4 Laboratory of Epigenetics, Institute for Protein Research, Osaka University, Suita, Osaka, Japan; The University of Adelaide, Australia

## Abstract

Dental pulp cells (DPCs) are known to be enriched in stem/progenitor cells but not well characterized yet. Small non-coding microRNAs (miRNAs) have been identified to control protein translation, mRNA stability and transcription, and have been reported to play important roles in stem cell biology, related to cell reprogramming, maintenance of stemness and regulation of cell differentiation. In order to characterize dental pulp stem/progenitor cells and its mechanism of differentiation, we herein sorted stem-cell-enriched side population (SP) cells from human DPCs and periodontal ligament cells (PDLCs), and performed a locked nucleic acid (LNA)-based miRNA array. As a result, miR-720 was highly expressed in the differentiated main population (MP) cells compared to that in SP cells. *In silico* analysis and a reporter assay showed that miR-720 targets the stem cell marker *NANOG*, indicating that miR-720 could promote differentiation of dental pulp stem/progenitor cells by repressing *NANOG*. Indeed, gain-and loss-of-function analyses showed that miR-720 controls NANOG transcript and protein levels. Moreover, transfection of miR-720 significantly decreased the number of cells positive for the early stem cell marker SSEA-4. Concomitantly, mRNA levels of DNA methyltransferases (*DNMTs*), which are known to play crucial factors during stem cell differentiation, were also increased by miR-720 through unknown mechanism. Finally, miR-720 decreased DPC proliferation as determined by immunocytochemical analysis against ki-67, and promoted odontogenic differentiation as demonstrated by alizarin red staining, as well as alkaline phosphatase and osteopontin mRNA levels. Our findings identify miR-720 as a novel miRNA regulating the differentiation of DPCs.

## Introduction

Stem cells are undifferentiated cells characterized by their ability for self-renewing division as well as by their capacity into differentiate to other cell types [1], [2]. The identification and characterization of adult stem cells in various tissues has led to a greater understanding of development, tissue maintenance and self-renewal. Deeper understanding of the biology of stem cells would offer great promise in the field of regenerative medicine.

MicroRNAs (miRNAs) have emerged as important regulators of stem cell maintenance and function [3], [4]. miRNAs are a class of small noncoding RNAs of approximately 17 to 25 nucleotides that mainly regulate protein translation by recognizing the 3′-untranslated region (UTR) of their target mRNAs [5], [6], [7], [8]. Some miRNAs are expressed in tissue-specific and/or developmentally regulated manners, and are crucial for maintaining the balance between proliferation and differentiation during development. Following studies showing that a set of transcription factors induces pluripotent stem cells [9], transfection of mature miRNAs has also been reported to reprogram differentiated fibroblasts to pluripotency [10], [11]. In addition, previous studies have demonstrated the involvement of miRNAs (e.g., miR-30d, miR-138, miR-155, miR-18a) in the process of osteogenic or adipogenic differentiation of bone marrow stem/progenitor cells (BMSCs) [12], [13], [14]. Therefore, miRNAs play important roles in the determination of stem cell fate.

In this study, we have attempted to identify a miRNA signature involved in regulating the maintenance of stem cell phenotype or differentiation ability of dental tissue-derived stem/progenitor cells. Our hypothesis is that stem cells can be regulated by specific miRNAs that orchestrate the translation of mRNAs related to cell differentiation or the maintenance of stem cell phenotype [15]. A main drawback in such studies, however, is related to the absence of a specific marker for isolation of stem cells from adult mesenchymal tissues. In order to overcome such hindrance, we have based our strategy on the properties of stem cells to efflux Hoechst-33342 dye through ATP binding cassette (ABC) transporters [16], [17]. Such cells have been described as side population (SP) cells and are enriched in stem cells [17], [18]. We thus have sorted SP cells and main population (MP) cells from dental pulp cells (DPCs) as well as periodontal ligament cells (PDLCs) by fluorescence-activated cell sorting (FACS), and then performed a miRNA array analysis. Subsequent experiments revealed that miR-720 directly control the levels of NANOG, a key transcription factor and marker of stem cells, and differentiation of DPCs.

## Materials and Methods

### 2.1. Cells

DPCs and PDLCs were isolated from third molars or pre-molars extracted from at least 4 adults under the approved guidelines and protocol (Okayama University Ethics Committee #418) with written informed consent obtained from all subjects. The isolation and cultivation of human DPCs and PDLCs were performed according to a previously reported method [19], [20]. Cells were cultured in α-Modified Essential Medium (α-MEM, Life Technologies^TM^,Carlsbad,CA, USA) supplemented with 15% fetal bovine serum (FBS; Life Technologies^TM^), 100 mM L-ascorbic acid 2-phosphate (Wako Pure Chemical Industries, Osaka, Japan), 2 mM L-glutamine (Life Technologies^TM^), 100 units/ml penicillin (Sigma, St Louis, MO, USA) and 100 μg/ml of streptomycin (Sigma) at 37°C under 5% CO_2_ in air.

HeLa cells were cultured in high glucose Dulbecco's Modified Eagle Medium (DMEM, Life Technologies^TM^) supplemented with 1% antibiotics and 10% FBS.

### 2.2. Flow cytometry (FCM) and Fluorescence-activated cell sorting (FACS)

DPCs and PDLCs were dissociated with accutase (Innovative Cell Technologies Inc., San Diego, CA, USA) and filtered through a 70-μm cell strainer, washed with phosphate buffer saline (PBS), resuspended in 1% FBS/PBS, and incubated with antibodies against human SSEA-4, CD29, CD34, CD44, CD45 and CD146 (BD Biosciences, San Jose, CA, USA) for 30 min on ice. Cells were then washed, and subjected to a FCM analysis by MACSQuant® Analyzer (Miltenyi Biotec, Bergisch Gladbach, Germany) or Accuri™ C6 (BD Biosciences). Gating of positive cells was established as the histogram gate giving 1% of positive cells using the corresponding isotype control.

For cell sorting, approximately 1×10^8^ DPCs or PDLCs were dissociated with accutase and stained with Hoechst-33342 (5 μg/mL, Sigma) according to a previously described protocol [17]. Verapamil (100 μM, Sigma) was used to inhibit the efflux of the dye by ABCG2 transporters and for gating of SP cells. Stained cells were analyzed and sorted on a cell sorter (FACSAria™ II, BD Biosciences) by a 375 nm near ultra-violet laser and detected by Hoechst red (675 nm Long pass) and Hoechst blue (430–470 nm) filters. Sorted cells were immediately frozen at −80°C for subsequent RNA isolation for the miRNA array, or aliquoted in a small quantity for subsequent colony forming unit-fibroblast (CFU-F) assay.

### 2.3. Colony forming unit assay

CFU-F assay was performed by seeding 100 cells in a 25-cm^2^ culture flasks and culturing for 2 weeks. Cells were then washed with PBS and stained with 0.1% toluidine blue contained in 1% PFA overnight. On the following day, cells were washed to remove excess dye. Stained clusters containing more than 50 cells were counted as positive colonies.

### 2.4. MicroRNA array

A miRNA array was performed with total RNA collected with RNAzol RT (Molecular Research Center, OH, USA) from single samples of SP and MP cells sorted from both DPCs and PDLCs on a locked nucleic acid (LNA) platform (miRCURY LNA™ microRNA Array, Exiqon Life Sciences, Vedbaek, Denmark). A total of 250 ng of total RNA from MP cells was used for microRNA array. In the case of SP cells, since there was not satisfactory amount of RNA due to the extremely low number of sorted cells (0.1%), a total of 3 μL was applied for the array. RNA isolation, array and clustering analyses were performed by the manufacturer. Briefly, array protocol consisted of sample labeling with Hy3 fluorescent label, purification with 3 M NaOAc and isopropanol, hybridization, wash, scan and data analysis. Average of signal intensity in 4 repeated spots in the array was considered as the final data for each probe. Probes with a percent coefficient of variation (%CV, standard deviation/mean×100) above 50% were excluded. Raw data can be accessed at gene expression omnibus (GEO) database (accession number GSE47025).

Hierarchical clustering was performed by average linkage measurement and Pearson's correlation metric in the freely-available, open-source MeV software tool available from http://www.tm4.org/mev/. A comparative analysis of miRNA expression profile between SP cells or MP cells from DPCs and PDLCs was performed in order to investigate the effects of isolation lots and concentrations of RNA. Additionally, analysis of gene expression levels of *ABCG2* transporter and the stem cell markers *NANOG* and *OCT-4* between SP and MP cells was performed using residual RNA.

### 2.5. Reverse transcription and real-time reverse transcription-polymerase chain reaction (RT-PCR)

Total RNA from DPC cultures was extracted with miRNeasy (Qiagen, Hilden, Germany) and purified by removing genomic DNA with RNase-Free DNase set (Qiagen), as described previously [14], [21]. Primer sequences are shown in Table 1. Gene expression levels were normalized to that of ribosomal protein S29.

**Table 1:** List of primer pairs used for real time RT-PCR analysis.

Gene name (accession #)	Direction	Nucleotide Sequence
*S29* (BC032813)	Sense	5′-TCTCGCTCTTGTCGTGTCTGTTC-3′
	Anti-sense	5′-ACACTGGCGGCACATATTGAGG-3′
*NANOG* (NM_024865.2)	Sense	5′-GCCTTCACACCATTGCTAT-3′
	Anti-sense	5′-TCTCCAACATCCTGAACCT-3′
*OCT-4* (NM_001159542.1)	Sense	5′-GAAAGGGACCGAGGAGTA-3′
	Anti-sense	5′-CCGAGTGTGGTTCTGTAAC-3′
*ALP* (NM_000478)	Sense	5′- GCACCGCCACCGCCTACC-3′
	Anti-sense	5′- CCACAGATTTCCCAGCGTCCTTG-3′
*OPN* (BC007016)	Sense	5′- ATGTGATTGATAGTCAGGAACTT-3′
	Anti-sense	5′- GTCTACAACCAGCATATCTTCA-3′
*ABCG2* (NM_001257386.1)	Sense	5′- CATTCAAGAGTTAGGTCTGGATAA-3′
	Anti-sense	5′- CCAAGAACAAGATGGAAGGAT-3′
*DNMT3A* (NM_175629.2)	Sense	5′-GCAGCCATTAAGGAAGAC-3′
	Anti-sense	5′-TGGTTATTAGCGAAGAACATC-3′
*DNMT3B* (NM_006892.3)	Sense	5′-TTACCTTACCATCGACCTCACA-3′
	Anti-sense	5′-CTGTCTCCATCTCCACTGTCT-3′
*DNMT1* (NM_001379.2)	Sense	5′-CCATCAGGCATTCTACCA-3′
	Anti-sense	5′-CGTTCTCCTTGTCTTCTCT-3′
miR-720		5′-TCTCGCTGGGGCCTCCA-3′

For analysis of miRNA levels, complementary DNAs were obtained using a miR-X miRNA First-Strand Synthesis Kit (Clontech, Mountain View, CA, USA) from 1 μg of total RNA, according to the manufacturer's instructions. Expression levels of miRNAs were quantified by real-time RT-PCR using SYBR green (Clontech) and normalized to that of the internal control U6.

### 2.6. *In silico* target prediction

Targets of the selected miRNAs were predicted by utilizing miRDB software (http://mirdb.org/miRDB). Possible complementary sequences of miR-720 in *NANOG* mRNA sequence were searched using RegRNA software (http://regrna.mbc.nctu.edu.tw/html/prediction.html) [22].

### 2.7. Reporter plasmid constructs

For target validation, the reporter gene construct containing 3 tandem copies of the *in silico-*determined miR-720 predicted target site in *NANOG* 3′-UTR was constructed by inserting the corresponding synthetic oligodeoxynucleotides between the XbaI-EcoRI restriction sites at the 3′-UTR of *luciferase* in a recipient pGL3L(+) reporter vector [23]. Additional oligodeoxynucleotides containing mutations in *NANOG* 3′-UTR seed sequence were designed, synthesized and also inserted into the reporter vector. Designed oligonucleotides sequences of the predicted sites are shown in Table S3. Final vector constructs were verified by DNA sequencing before transfection into HeLa cells.

### 2.8. Transient transfections

DPCs were transfected with hsa-miR-720 Mimic (*mir*Vana*®*, Life Technologies^TM^), miRNA Mimic Negative Control #1 (*mir*Vana™, Life Technologies^TM^), miR-720 inhibitor (*mir*Vana®, Life Technologies^TM^) or miRNA Inhibitor Negative Control #1 (*mir*Vana™, Life Technologies^TM^) using Lipofectamine® RNAiMAX (Life Technologies^TM^) transfection reagent, according to the manufacturer's instructions. Inhibitors and mimics were used at a concentration of 20 nM. Cells were collected 24 h after transfection for further analysis of mRNA or miRNA levels.

Transfection of luciferase reporter plasmids into HeLa cells was performed with Lipofectamine® 2000 (Life Technologies^TM^), according to manufacturer's protocol. DNMT3a and DNMT3b overexpression vectors, which were kind gifts from Dr. Stephen B. Baylin, were also transfected with Lipofectamine® 2000 into DPCs.

For co-transfection of miRNA and effector plasmid constructs, cells were first transfected with anti-miR-720 and cultured for 24 h. Subsequently, medium was changed and cells were transfected with the constructs and cultured for another 24 h before analyses.

### 2.9 Luciferase assay

HeLa cells were seeded in white opaque 96-well plates, transfected with luciferase reporter constructs and cultured for 8 h. Next, medium was changed, and cells were transfected with mimic-miR-720 and cultured for additional 24 h before assay. Luciferase assay was performed using Bright-Glo™ Luciferase Assay System (Promega), according to manufacturer's instruction.

### 2.10. Imaging and quantitative immunocytochemical analysis

DPCs were seeded in 96-well plates, transfected with anti-miR-720, mimic-miR-720 or the respective negative controls, and cultured for 24 h. Immunocytochemical analyses were performed according to methods described previously [21], using primary antibodies against DNMT1 [24], [25], DNMT3A [26], DNMT3B (Imgenex Corporation, San Diego, CA, USA), NANOG (ReproCELL, Boston, MA, USA) or isotype control IgG. Cell proliferation was determined by the number of cells positive for the proliferation marker ki-67 using anti-human ki-67 antibody (Abcam, Cambridge, UK), as previously reported [21]. Alexa Fluor® 488-conjugated antibody (Life Technologies^TM^) was utilized as secondary antibody. Cell nuclei were stained with 4′ 6-diamidino-2-phenylindole (DAPI, Life Technologies^TM^). Images of the cells were acquired and quantitatively analyzed using an automated fluorescence imaging system (Cellomics ArrayScanTM VTI high content screening reader, Thermo Scientific, Waltham, MA, USA).

### 2.11. Analysis of odontogenic differentiation *in vitro*

DPCs were seeded in 24-well plates, and transfected with anti-miR-720, mimic-miR-720 or the respective negative controls, and cultured until confluency. Cells were then induced to differentiate toward odontogenic lineage under odontogenic-inducing medium, as previously described [14].

Alkaline phosphatase staining was performed with nitro-blue tetrazolium and 5-bromo-4-chloro-3′-indolyphosphate (NBT-BCIP, Roche, Basel, Switzerland), and alizarin red staining for Ca^+^ deposition was performed with 1% Alizarin red S (Sigma) solution (pH 6.4), as described previously [14].

### 2.12. Statistical analysis

Statistical analyses were performed by unpaired Student's *t*-test or one-way ANOVA followed by Tukey post-hoc correction tests when appropriate.

## Results

### 3.1. Characterization, sorting of DPCs and PDLCs, and microRNA analysis

**Figure 1:**
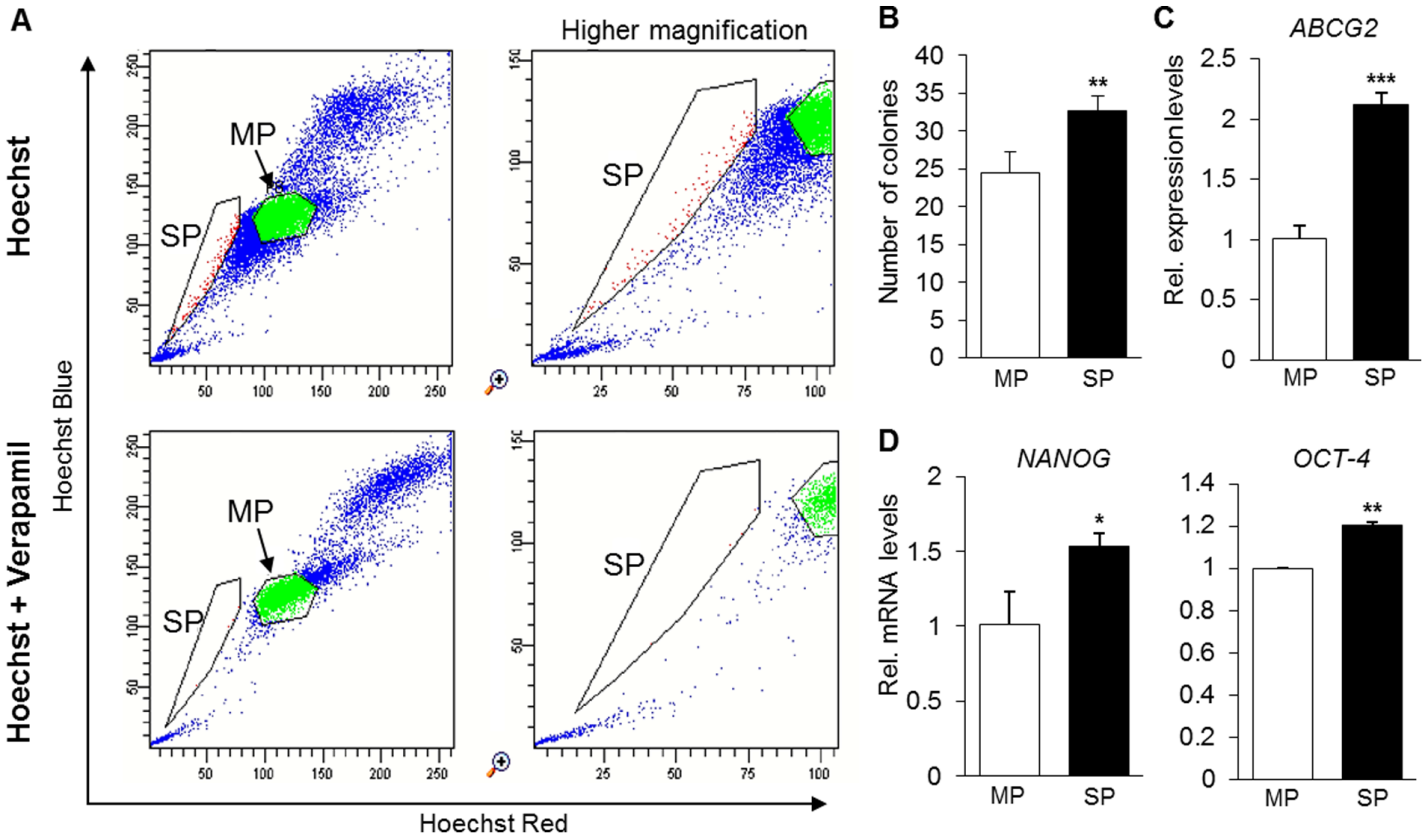
Sorting and characterization of MP and SP cells from DPCs. A) Identification and sorting of side population (SP) and main population (MP) by FACS using Hoechst-33342 (5 μg/mL) and verapamil (100 μM) as an inhibitor of ABCG2 binding cassette. B) Quantitative analysis of colony forming ability (CFU-F assay) of SP and MP cells. Results are the mean (±S.E.M.) of quadruplicate samples. C–D) mRNA levels of *ABCG2*, *NANOG* and *OCT-4* in SP and MP cells. Results are the average (±SD) of a single experiment run in triplicate. * P<0.05, ** P<0.01, *** P<0.001, unpaired *t*-test, compared to MP.

Firstly, in order to characterize the whole population of DPCs, the expression of stem cell surface markers was analyzed by FCM. As a result, DPCs were shown to be positive for mesenchymal stem cell markers SSEA-4 [27], CD146 [28] and CD44 [29], and negative for hematopoietic stem cell markers CD24, CD34 and CD45 (Fig. S1). Next, DPCs were sorted in order to isolate MP and SP cells, which corresponded to only a small fraction of 0.1% of the total number of sorted cells (Fig. 1A). The results of CFU-F assay using sorted MP and SP cells demonstrated a higher capacity of SP cells to form colonies (Fig. 1B). In addition, using residual RNA that remained after the array, we analyzed the expression levels of the ABCG2 transporter. As expected, SP cells showed a significantly higher expression of the ABCG2 transporter, a finding which is consistent with the higher capacity of the cells to efflux Hoechst dye (Fig. 1C). Additionally, since the SP cohort is known to be enriched in stem cells, we also investigated and confirmed the higher expression levels of two stem cell markers, *NANOG* and *OCT-4* in SP cells (Fig. 1D). Taken together, these data demonstrate that the SP of DPCs presents higher stem cell properties than MP cells.

**Figure 2:**
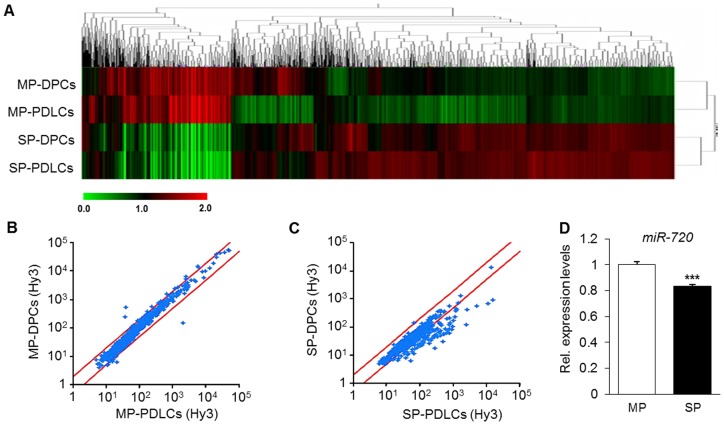
miRNA expression signature in DPCs and in periodontal ligament-derived cells (PDLCs). A) Clustering analysis of SP and MP cells from DPCs and PDLCs. B) A comparative scatter plot analysis of miRNA profiles in MP from DPCs and PDLCs. C) A comparative scatter plot analysis of miRNA profiles in SP from DPCs and PDLCs. Red line shows variations of 2 fold (upper line) and 1/2 fold (lower line) change. D) Quantification of miR-720 expression level in sorted MP and SP cells. Results are the average (±SD) of a single experiment run in triplicate. *** P<0.001, unpaired *t*-test, compared to MP.

We next carried out miRNA expression profiling on DPCs and PDLCs. Interestingly, the results of the clustering analysis of the miRNA array revealed a similar profile between DPCs and PDLCs (Fig. 2A). Additionally, comparative analysis of miRNA expression profiles between the MP cells or SP cells of DPCs and PDLCs also showed that the sorted cell populations presented a good consistency between DPCs and PDLCs, which strengthen the reproducibility of the methods and results (Fig. 2B and 2C). A high reproducibility was observed in MP cells between the two cell types (Fig. 2B); however, since the amount of collected SP cells was extremely low, the total amount of RNA isolated was not enough to enable more consistent results between SP cells from DPCs and PDLCs. Therefore, additional normalization (with 75% tiling) was performed in order to minimize effects which were not due to the controlled factors in the experiment.

The complete list of miRNAs differentially expressed in MP and SP cells is presented in Table S1 and S2, respectively. Among the whole miRNA profile, miR-1260b and miR-720 were the two most highly expressed miRNAs in MP cells, whereas miR-200b and miR-607 were highly expressed in SP cells.

### 3.2. Target prediction and confirmation of array data

We next investigated the predicted targets *in silico* of the 6 most highly expressed miRNAs in MP and SP cells as shown in Table 2 and 3, respectively. Of particular interest, miR-720 was predicted to target only 22 candidate genes, among which two genes has been reported to play important roles in the biology of stem cells, namely *DNMT3A* and *NANOG*. Before proceeding to further experiments, we therefore analyzed the expression level of miR-720 in MP and SP cells. As shown in Fig. 2D, expression of miR-720 was lower in SP cells, suggesting that miR-720 could be involved in the regulation of stemness and/or differentiation of DPCs.

**Table 2:** *In silico* target prediction analysis of the 6 most highly expressed miRNAs in MP cells.

	Name	Predicted targets
1	miR-1260b	DNMT3A, HOXD1, OCT1, FGF12, GDF11
2	miR-720	DNMT3A, NANOG
3	miR-1280	JAG2, ZNF544, UBTF
4	miR-491-3p	EGFR, SOX-11, COL12A1, NOV/CCN3
5	miR-1260a	DNMT3A, PDGFD, HOXD1, SEMA3A, FGF12
6	miR-138-1	TIEG2, JMJD1C, CYCLIN D3, ROCK2, PPARδ

Table shows the selected possible mRNA targets related to stemness and cell differentiation.

**Table 3:** *In silico* target prediction analysis of the 6 most highly expressed miRNAs in SP cells.

	Name	Predicted targets (miRDB)
1	miR-200b	ZEB1, ZEB2, BMI1, TWISTNB, PLEXINA4, DNMT3A, CDNK2B
2	miR-607	RUNX-1, RUNX-2, IGF-1, MSX1, SOCS4, CXCL6
3	miR-515-5p	FGFR2, TGFBR2, NOTCH2, BMP8b, MSX2, FGF12, BMPR1B
4	miR-1245	FGF20, TBRG1, BMI1
5	miR-3919	NFAT5, ID4, HOXB13, HDAC1, RPS6KA6, TGFBR2, SEMA3A
6	miR-182	FGF9, SMAD1, IGF1R, SOX2, BMPR1B, WISP1/CCN4, EGR3

Table shows the selected possible mRNA targets related to stemness and cell differentiation.

### 3.3. miR-720 controls the expression of stem cell markers in DPCs

**Figure 3:**
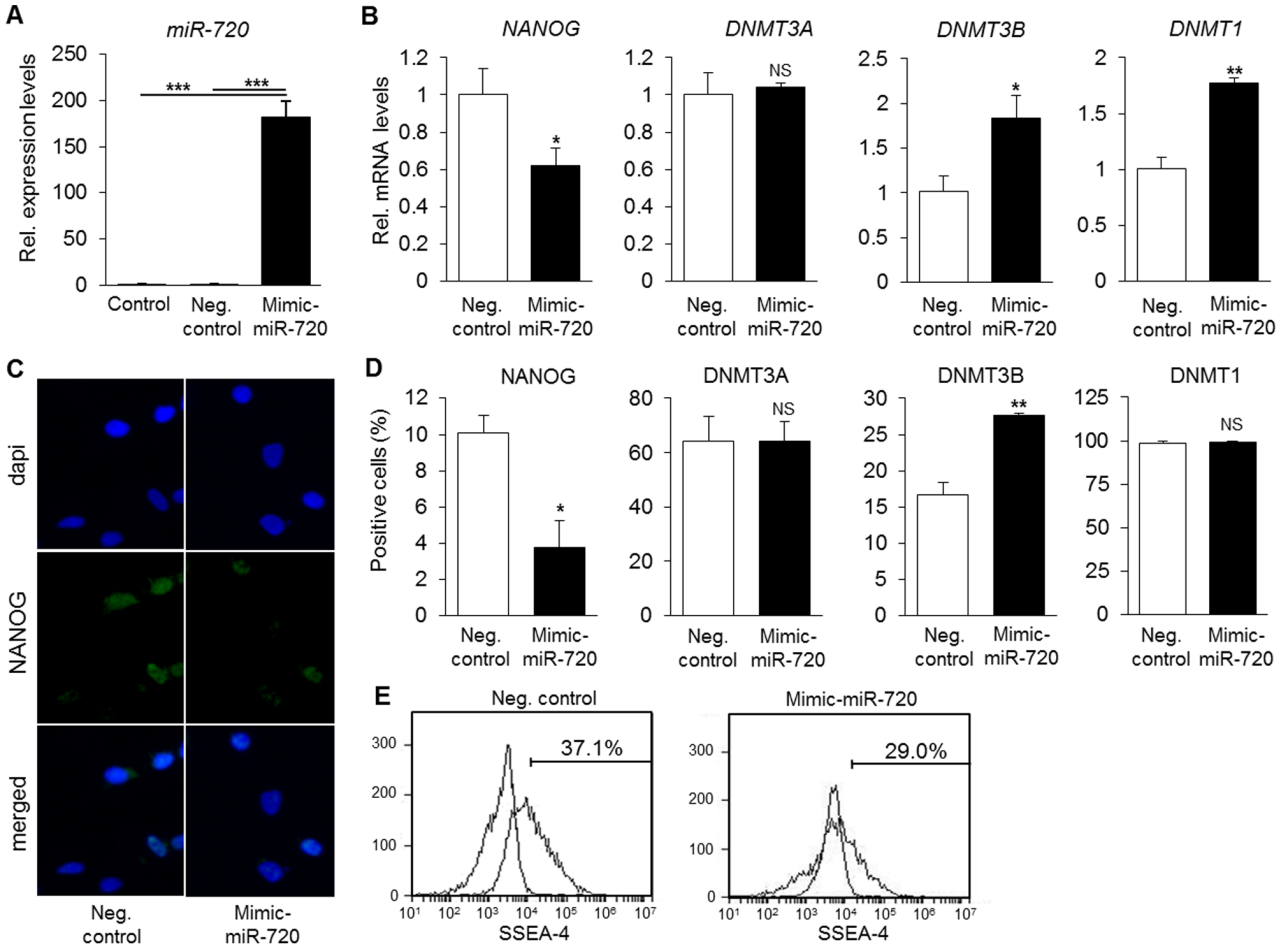
Effects of mimic-miR-720 on stem cell phenotype of DPCs. A) Quantitative assessment of miR-720 levels. *** P<0.001, One way ANOVA/Tukey. Results are representative of at least 3 independent experiments. B) mimic-miR-720 reduced mRNA levels of *NANOG*, but increased the levels of *DNMT3B* and *DNMT1* mRNA. No significant changes were observed in *DNMT3A* mRNA upon miR-720 transfection. * P<0.05, ** P<0.01, NS =  non-significant, unpaired *t*-test, compared to si-control group. Results are representative of at least 3 independent experiments. C) Representative images of immunocytochemistry for NANOG protein levels. D) Quantitative analysis of NANOG or DNMTs positive cells. Mimic-miR-720 significantly reduced the number of NANOG positive cells, while increasing those for DNMT3B. There was no significant change in the percentages of DNMT1 or DNMT3A positive cells. Results in C–D are representative of at least 3 independent experiments. Quantitative analysis was performed on 500 cells/well, with at least triplicate samples. * P<0.05, ** P<0.01, NS =  non-significant, unpaired *t*-test, compared to si-control group. G) Transfection of mimic-miR-720 decreased the percentage of SSEA-4^+^ cells. Results are representative of 3 independent experiments.

In order to address whether miR-720 can regulate the levels of NANOG and DNMT3A, we performed gain- and loss-of-function experiments on miR-720 and analyzed alterations in the levels of the two potential target genes. In addition, we also analyzed the expression levels of DNMT3B and DNMT1, as well as the expression of the surface protein SSEA-4, which has been reported to be a marker of stem/progenitor cells from dental pulp [27]. As shown in Fig. 3, mimic-miR-720 in DPCs significantly decreased the levels of *NANOG* mRNA, while increasing the expression levels of *DNMT3B* and *DNMT1* (Fig. 3B). However, minimal changes were observed in the levels of *DNMT3A* mRNA. In agreement, immunocytochemical analysis also showed a decrease in the number of cells positive for NANOG (Fig. 3D). Consistent with a decrease in the levels of *NANOG*, there was also a decrease in the percentage of SSEA-4^+^ cells (Fig. 3G). On the other hand, DNMT3B levels were significantly increased upon transfection of mimic-miR-720 (Fig. 3D), whereas DNMT1 and DNMT3A protein levels were not significantly changed.

**Figure 4:**
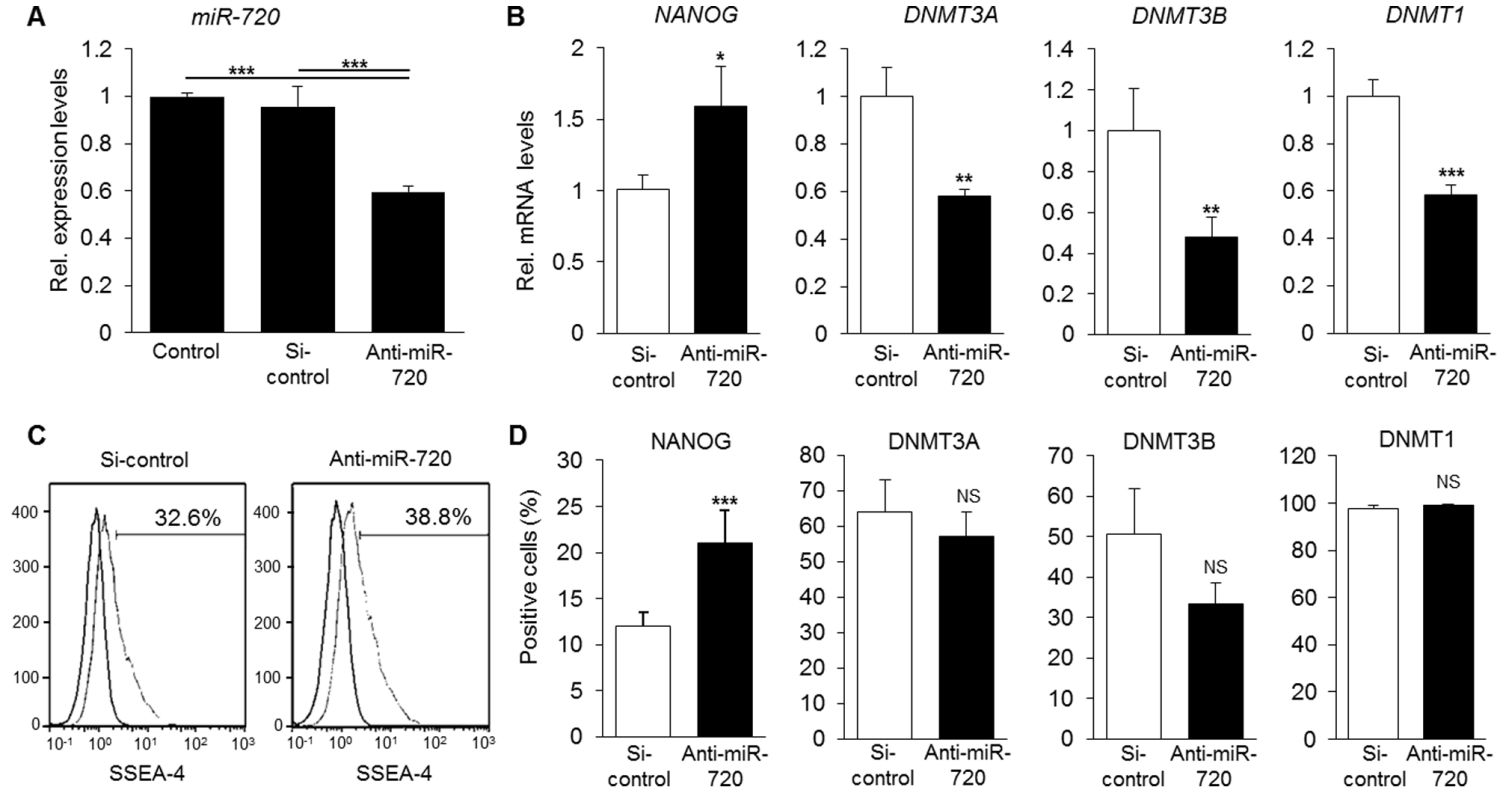
Effects of miR-720 blockade on stem cell phenotype of DPCs. A) Quantitative assessment of the knockdown efficiency of miR-720. *** P<0.001, One way ANOVA/Tukey. Results are representative of at least 3 independent experiments. B) Knockdown of miR-720 increased the levels of *NANOG* transcripts, but reduced significantly the levels of *DNMT3A*, *DNMT3B* and *DNMT1* transcripts. * P<0.05, ** P<0.01, *** P<0.001, unpaired *t*-test, compared to si-control group. Results are representative of at least 3 independent experiments. C) Knockdown of miR-720 increased the percentage of cells positive for SSEA-4. Results are representative of 3 independent experiments. D) Quantitative immunocytochemical analysis of NANOG, and DNMTs positive cells upon knockdown of miR-720. Anti-miR-720 increased the percentage of NANOG positive cells, but induced no significant changes in the percentage of DNMTs positive cells. NS =  non-significant, unpaired *t*-test, compared to si-control group. Results are representative of at least 3 independent experiments. Quantitative analysis was performed on 500 cells/well, at least with triplicate samples.

We further proceeded to loss-of-function analysis, and we could observe that miR-720 knockdown induced a significant increase in the number of cells positive for NANOG (Fig. 4B, D), as well as in the number of SSEA-4^+^ cells (Fig. 4C). Conversely, knockdown of miR-720 induced a significant decrease in *DNMT3A*, *DNMT3B* and *DNMT1*, mRNA expression which however, did not correspond to alterations at a protein level (Fig. 4B and 4D). Taken together, these data demonstrated that miR-720 can regulate the expression of stem cell markers in DPCs. On the other hand, since increases in the levels of miR-720 correlated with those of DNMTs, we ruled out the possibility of a direct regulation of these genes by miR-720 through direct binding to their 3′-UTR, and proceeded with further target validation only with NANOG.

### 3.4. miR-720 recognizes *NANOG* 3′-UTR

**Figure 5:**
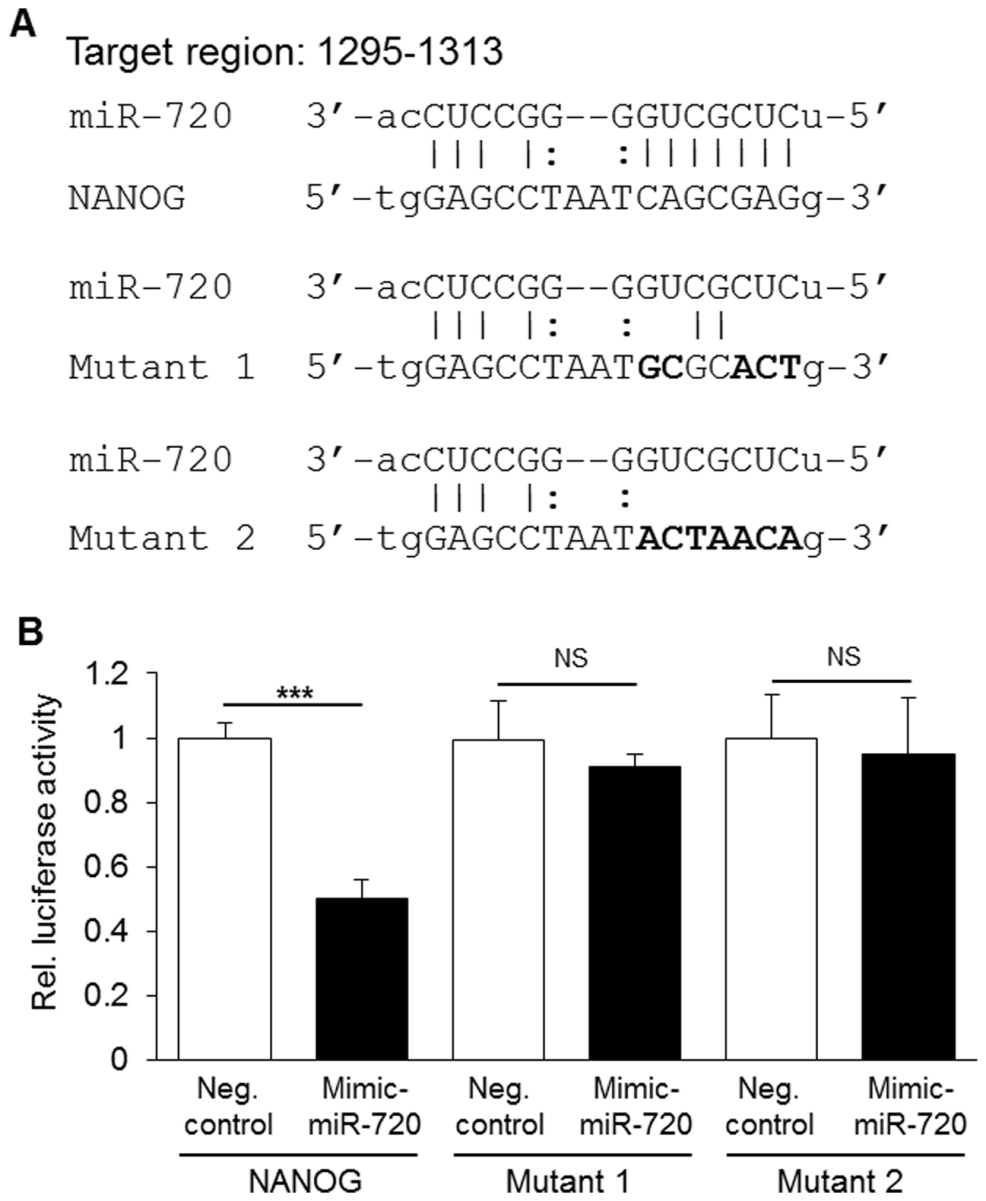
Identification of a miR-720 target site in *NANOG* mRNA. A) *In silico* analysis of putative target region of miR-720 in the mRNA sequence of *NANOG*, and mutations in the seed sequence of *NANOG* 3′-UTR. The target region indicates number from 5′ end of the mRNA. Bars indicate Watson-Crick base pairing. Dots indicate G–U non-Watson-Crick base pairing. Mutations are shown in bold letters. B) Validation of the putative miR-720 target site. A triple tandem repeat of the putative miR-720 site or mutants were cloned into luciferase reporter construct and assayed in HeLa cells. miR-720 significantly decreased luciferase activity of the wild type reporter plasmid with the miR-720 recognition site in *NANOG* 3′-UTR, whereas no significant difference was observed in the mutants. Results are mean (± S.E.M.) of 3 independent experiments performed at least with quadruplicate wells. *** P<0.001, NS =  non-significant, unpaired *t*-test.

In an attempt to clarify the mechanisms involved in miR-720 regulation of NANOG, we performed an *in silico* search for possible miR-720 recognition sequences in the *NANOG* 3′-UTR, identifying in the process a single putative target region (Fig. 5A). In order to determine whether this putative miR-720 recognition sequence was functional, we then designed and constructed a triple tandem repeat of the sequence, and the mutants (mutant 1 and mutant 2), and cloned into a luciferase reporter plasmid (Fig. 5A and table S3). As shown in Fig. 5B, a significant decrease in luciferase activity was observed with the wild type mir-720 site of *NANOG* 3′-UTR upon mimic-miR-720 transfection, whereas no significant changes were observed with mutants. These results clearly indicate that miR-720 can recognize the specific miR-720 site in the *NANOG* 3′-UTR and that miR-720 can inhibit translation and stability of *NANOG* transcripts.

### 3.5. Control of *NANOG* transcripts by miR-720 is partially mediated by DNMT3A and DNMT3B

**Figure 6:**
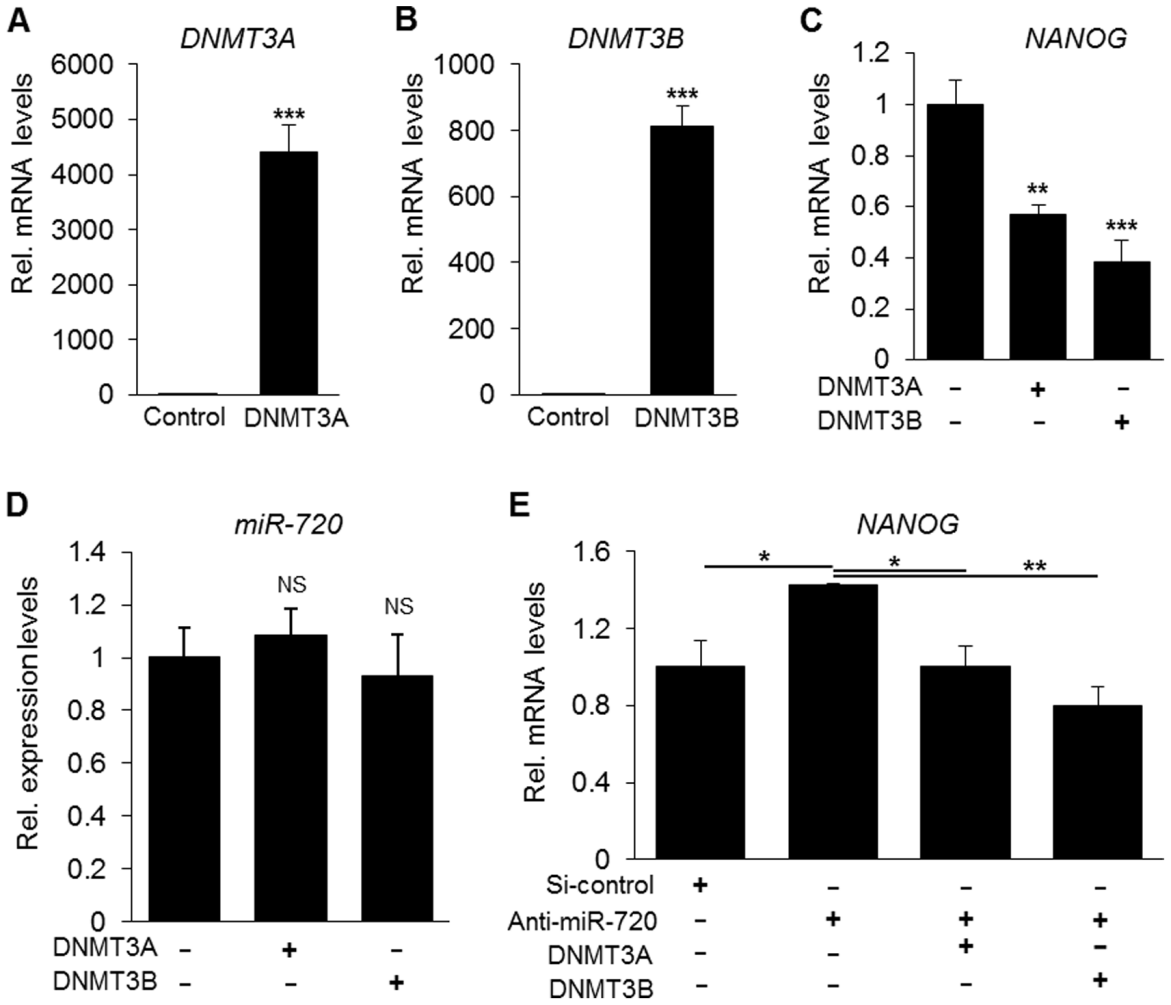
Effects of DNMT3A and DNMT3B on miR-720 and *NANOG* expression levels. A–B) Overexpression of *DNMT3A* and *DNMT3B* upon transfection in DPCs cells. *** P<0.001, unpaired *t*-test, compared to pcDNA control group. Results are representative of 3 independent experiments. C–D) Quantification of *NANOG* mRNA and endogenous mature miR-720 upon overexpression of DNMT3A or DNMT3B. Either DNMT3A or DNMT3B reduced *NANOG* mRNA, but no significant changes were observed in miR-720 levels. Results in C–D are representative of 4 independent experiments. ** P<0.01, *** P<0.001, NS =  non-significant, one-way ANOVA/Tukey, compared to pcDNA control group. E) Quantification of *NANOG* mRNA upon co-transfection of anti-miR-720 and DNMT3A or DNMT3B overexpression constructs in DPCs. *NANOG* mRNA level was increased by miR-720 knockdown but was reduced by either DNMT3A or DNMT3B. Results are representative of 4 independent experiments. * P<0.05, ** P<0.01, one-way ANOVA/Tukey.

Based on previous reports that demonstrated that DNMT3A and DNMT3B act as transcriptional repressors of *Nanog* and *Oct-4* genes in embryonic stem (ES) cells [30], and that miR-720 increased *DNMTs* mRNA levels, we hypothesized that miR-720 could also control NANOG levels through an indirect mechanism mediated by DNMTs. In order to clarify this hypothesis, we firstly confirmed DNMT3A or DNMT3B overexpression upon plasmid transfection into DPCs (Fig. 6A–B). We then analyzed whether overload of DNMT3A or DNMT3B could repress *NANOG* gene expression in DPCs, in a similar manner as reported in ES cells. As expected, overexpression of DNMT3A or DNMT3B significantly reduced *NANOG* mRNA levels without alterations in the levels of miR-720 (Fig. 6C–D), which indicates that DNMT3A and DNMT3B repress *NANOG* expression, through a mechanism independent of miR-720. Finally, we performed co-transfection experiments with anti-miR-720 and DNMTs overexpression vectors, and observed that either DNMT3A or DNMT3B nullified the induction of *NANOG* transcript by miR-720 knockdown (Fig. 6E). Collectively, these results suggested that control of NANOG by miR-720 is partly mediated by DNMT3A and DNMT3B, which however work through a miR-720-independent pathway.

### 3.6. miR-720 regulates differentiation and proliferation of DPCs

**Figure 7:**
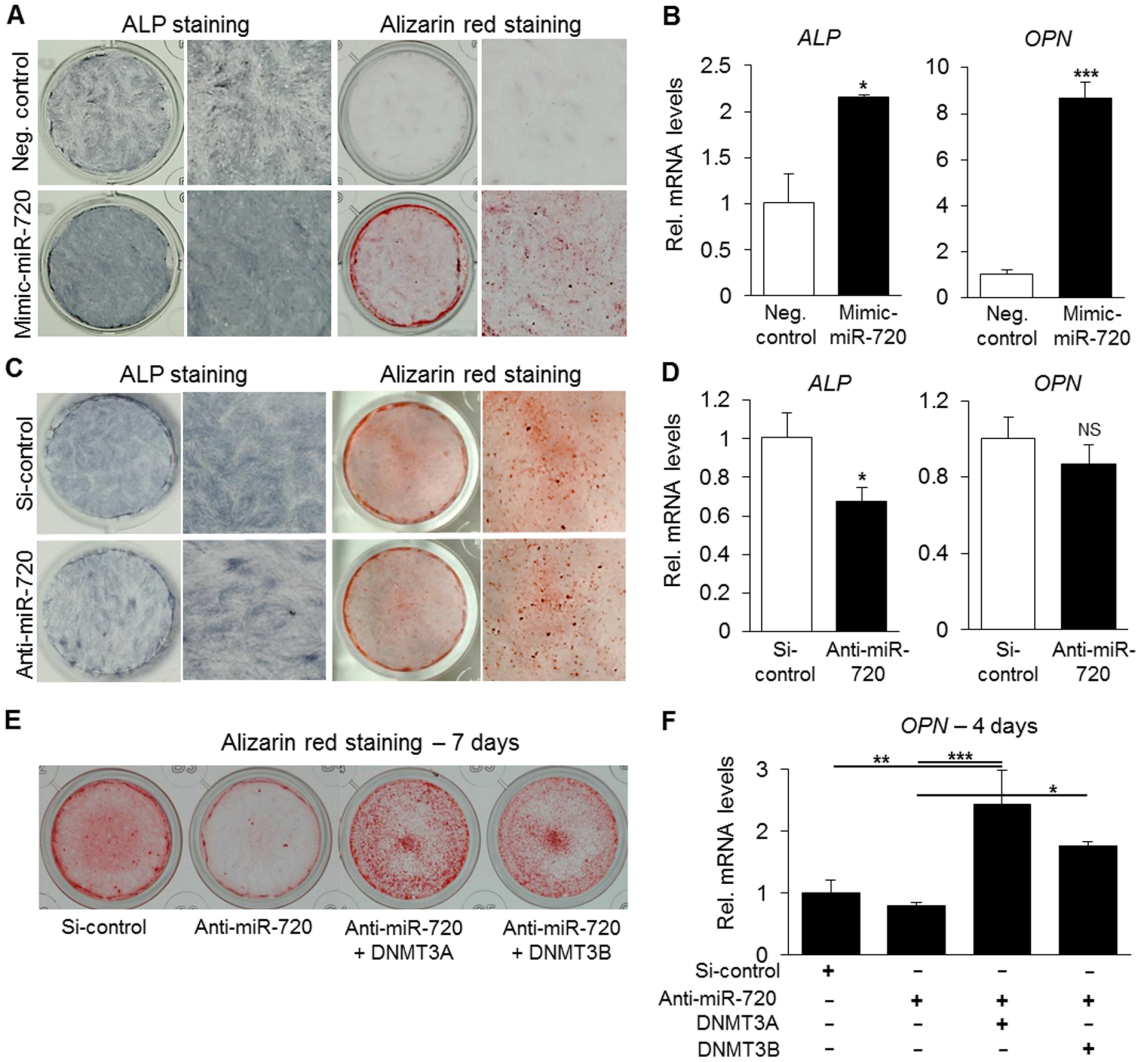
miR-720 promotes odontogenic differentiation of DPCs. A–B) Transfection of mimic-miR-720 promoted odontogenic differentiation of DPCs as demonstrated by ALP staining and alizarin red staining (A), as well as by mRNA quantification of *ALP* and *OPN*, mineralization markers. Results are representative of 3 independent experiments. * P<0.05, *** P<0.001, unpaired *t*-test, compared to si-control group. C–D) Knockdown of miR-720 decreased the differentiation of DPCs. Lower ALP activity and transcript levels were observed in anti-miR-720-transfected group. No significant changes in Alizarin red staining and *OPN* mRNA. Results are representative of at least 3 independent experiments. * P<0.05, NS =  non-significant, unpaired *t*-test, compared to si-control group. E–F) Co-transfection experiments with anti-miR-720 and DNMT3A or DNMT3B expression constructs. Anti-miR-720 inhibited DPC differentiation, but either DNMT3A or DNMT3B over-rescued the decreased odontogenic differentiation of DPCs induced by miR-720 knockdown, as demonstrated by alizarin red staining and *OPN* mRNA quantification, at day 7 and day 4 after odontogenic induction, respectively. Results are representative of at least 2 independent experiments. * P<0.05, ** P<0.01, *** P<0.001, one-way ANOVA/Tukey.

In order to investigate the function of miR-720 on the differentiation capacity of DPCs, we induced the cells to differentiate along the odontogenic lineage upon transfection of mimic-miR-720 or anti-miR-720. As shown in Fig. 7A and 7B, mimic-miR-720 enhanced the differentiation of DPCs, as demonstrated by ALP staining, alizarin red staining and mRNA levels of *ALP* and *OPN* – mineralization markers. Conversely, as expected, knockdown of miR-720 inhibited differentiation of DPCs (Fig. 7C–D), which demonstrates that miR-720 regulates the differentiation of DPCs.

Since DNMTs are known be crucial factors in embryonic stem cell differentiation, we performed co-transfection experiments of DNMTs and anti-miR-720 and assessed calcium deposition and expression of osteogenic markers to investigate whether the differentiation of DPCs induced by miR-720 could also be mediated by DNMT3A and/or DNMT3B. Interestingly, as shown in Fig. 7E–F, transfection of DNMT3A or DNMT3B over-rescued the decrease in odontogenic differentiation of DPCs caused by miR-720 knockdown, as demonstrated by alizarin red staining and *OPN* mRNA levels.

**Figure 8:**
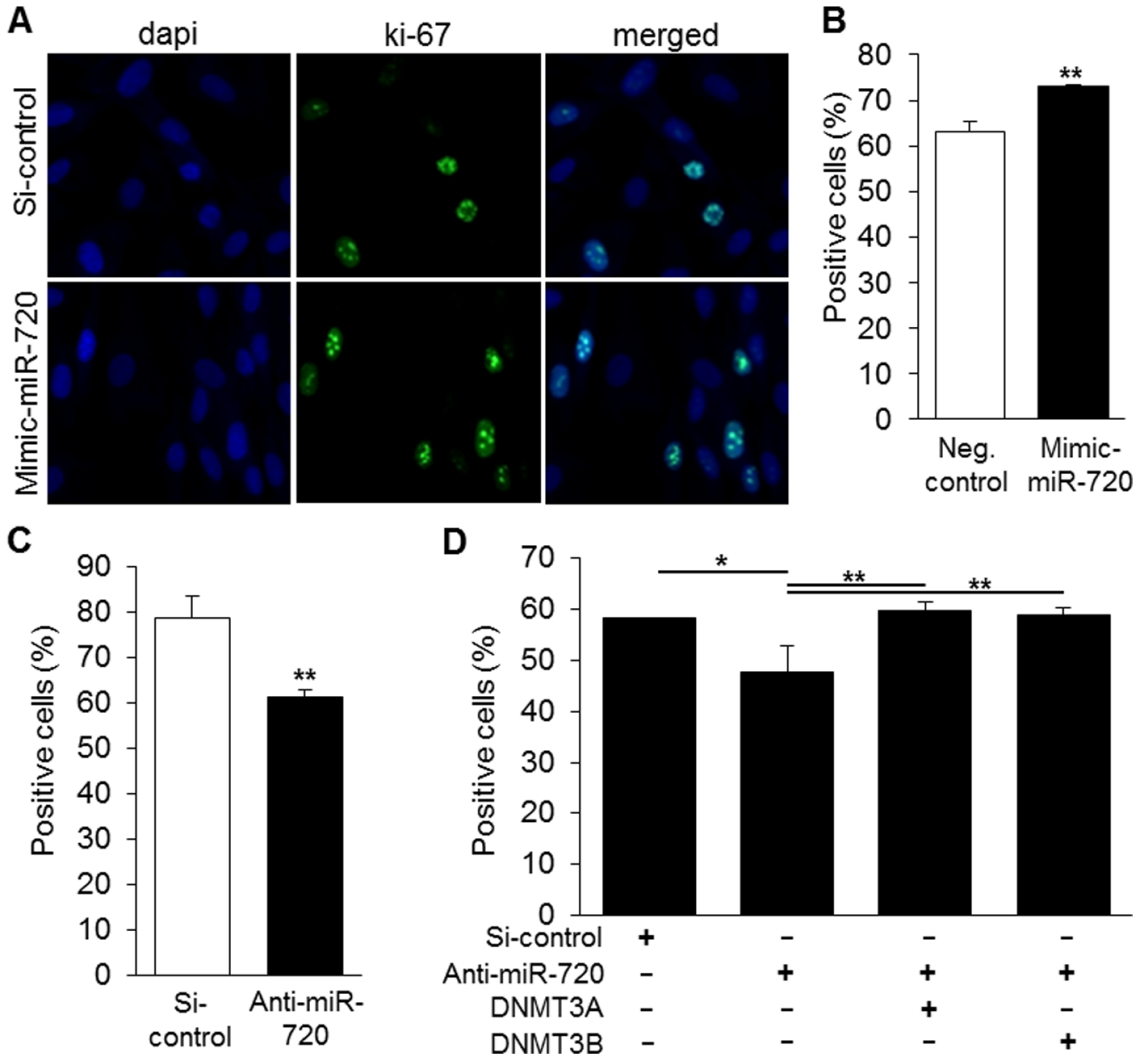
Cell cycle analysis upon transfection of mimic-miR-720 or anti-miR-720. A–B) Imaging and quantitative analysis of immunocytochemistry for ki-67, showing that miR-720 reduced proliferation of DPCs. Results are representative of 3 independent experiments. ** P<0.01, unpaired t-test, compared to si-control group. C) Knockdown of miR-720 increased the number of ki-67^-^ quiescent cells. ** P<0.01, unpaired *t*-test, compared to si-control group. Results are representative of at least 3 independent experiments. D) Co-transfection of anti-miR-720 and DNMT3A or DNMT3B expression constructs showed that either DNMT3A or DNMT3B nullified the decreased proliferation of DPCs induced by miR-720 blockade. Results are representative of at least 2 independent experiments. * P<0.05, ** P<0.01, one-way ANOVA/Tukey.

Finally, we analyzed the potential effects of miR-720 on the proliferation of DPCs by utilizing the cell proliferation marker ki-67, which is not expressed in G_0_ phase. As shown in Fig. 8, transfection of mimic-miR-720 increased the number of ki-67 positive cells (Fig. 8A, B), whereas miR-720 knockdown increased the number of ki-67 negative DPCs, i.e., quiescent cells (Fig. 8C). Co-transfection with anti-miR720 and/or DNMT3A or DNMT3B showed that either DNMT3A or DNMT3B could nullify the decreased proliferation of DPCs induced by miR-720 knockdown (Fig. 8D).

Taken together, these data demonstrate that miR-720 promotes cell cycle arrest, and odontogenic differentiation of DPCs. In addition, both DNMT3A and DNMT3B, which were also induced by miR-720, were shown to play important roles in promoting DPC differentiation through their intrinsic manner.

## Discussion

Epigenetic control of the mechanisms that govern stem cell fate by miRNAs has been regarded as a crucial component in stem cell biology. Previous studies have shown reprogramming of somatic cells to pluripotency by miRNAs alone (miR-200c, miR-302/miR-367/miR-369). In this context, the miR-200 and miR-302 families have been regarded as key factors in the maintenance and self-renewal of ES cell pluripotent state. On the other hand, miRNAs also control the differentiation of stem cells. Among several reported miRNAs, miR-134 has been shown to regulate the differentiation of murine ES cells to ectodermal lineages mainly by silencing *Nanog* and *Lrh1* [31]. miRNA-138 has also been reported to control osteogenic differentiation of BMSCs *in vitro* and *in vivo* [13].

An attractive feature of this study was that the process of stem cell isolation was based not on biomarkers of mesenchymal stem cells, but rather on their biological function. The ability of stem cells to efflux Hoechst 33342 has been demonstrated in a wide range of stem cell populations, including hematopoietic stem cells [32], cancer stem cells [33] or adult stem cells (including DPCs) [34]. In this study, we performed a miRNA array analysis on two different cell populations (DPCs and PDLCs), and despite the differences in their stem cell characteristics, we obtained similar miRNA signatures for each cell. Additionally, the results of the miRNA array showed that previously known miRNAs involved in pluripotency such as miR-302 and miR200c families were more highly expressed in SP cells (Table S2). On the other hand, miRNAs involved in the regulation of stem cell differentiation such as miR-138 and let-7 family RNAs were highly expressed in MP cells. Other studies based on the same strategy of SP cell sorting for isolation of stem cells have identified a set of miRNAs that regulate the stemness and differentiation of SP cells. For instance, miR-128a was highly expressed in SP cells, but its expression was decreased during differentiation of muscle SP cells [35]. Another study identified miR-328 in the SP of colorectal cancer cells and demonstrated that expression of miR-328 correlated with high a SP fraction, and that the main targets of miR-328 were the *ABCG2* transporter and the *MMP16* gene [36]. Finally, let-7 was found to be significantly down-regulated in SP cells, and decreased levels of let-7 were shown to promote SP cell differentiation. Consistently, our data showed similarly decreased expression of miR-128a and miR-328, and increased expression of let-7 family in MP of DPCs. Taken together, these data demonstrate that the methods applied in our study produced results highly consistent with prior works.

Since miR-720 levels were increased in differentiated MP cells, we suspected a possible association of miR-720 with the silencing of stem cell-related genes that could consequently enhance differentiation ability of cells. Indeed, mimic-miR-720 reduced the levels of NANOG as well as the percentage of SSEA-4^+^ cells and enhanced odontogenic differentiation of DPCs.

As demonstrated by luciferase assay, it is likely that miR-720 regulates *NANOG* transcripts by direct binding to the specific recognition site in its 3′-UTR (Fig. 5D). However, in addition, miR-720 overexpression was associated with increases in the levels of *DNMTs*, which is an unexpected effect of miRNAs. miRNAs are best known as repressors of translation and mRNA stability through base pairing to specific 3′-UTR target sites together with AGO and RISC protein complex. Thus, miR-720-driven increases in DNMTs expression could involve an indirect regulatory mechanism mediated by one or more unknown secondary factors. Alternatively, miR-720 could also target sequences in the promoters of *DNMT* genes and activate the transcription of the genes, similarly to the mechanisms reported in miR-373-induced E-cadherin *trans-activation* [22]. Further investigation will be necessary to clarify the exact mechanism behind these processes.

Previous reports also show that DNA methylation plays a central role in regulating the on/off switch of differentiation-related genes and signals. Additionally, dysfunctional methylation due to deficiencies in *Dnmt3a* and *Dnmt3b* has been reported to be associated with altered expression of *Nanog* and *Oct-4* during differentiation of murine ES cells. In these studies, DNMTs acted as transcriptional repressors of *Nanog* and *Oct-4* genes in ES cells [30], [37], [38]. Accordingly, we herein showed that DNMT3A and DNMT3B also repressed *NANOG* mRNA levels [30]; however, through a mechanism independent from the miR-720-mediated one (Fig. 7E).

Finally, a previous study has shown miR-720 transcribed downstream from the co-transcriptional factor Ski-related novel gene (SnoN/SKIL) in esophageal squamous cell carcinoma (ESCC) cells [39], and that down-regulation of SnoN decreased the levels of miR-720 as well as ESCC cell proliferation. Those results are in concord with our present experiments showing that miR-720 knockdown increases the number of non-proliferating ki-67 negative DPCs (Fig. 8).

Collectively, our data show that miR-720 participates in the control of the stem cell phenotype of DPCs by directly repressing NANOG levels and also indirectly, by enhancing the expression of DNMTs that can function as transcriptional repressing factors for *NANOG* gene. miR-720 also promoted DPC differentiation, and therefore, could be a potential target to either maintain the stem cell phenotype or to support bone/mineralized tissue formation in regenerative medicine.

## Supporting Information

Figure S1 **Characterization of DPCs by stem cell-related surface markers by FCM analysis.** DPCs were positive to SSEA-4, CD146 and CD44; and negative to CD24, CD34 and CD45.
(TIF)

Table S1 **List of miRNAs that presented more than 2 fold increase in MP cells.** Values show the fold change (MP/SP cells) in each cell type and the average. DPC =  dental pulp cells. PDLCs  =  periodontal ligament cells.
(XLS)

Table S2 **List of miRNAs that presented more than 1.15 fold increase in average in SP cells.** Values show the fold change (SP/MP cells) in each cell type and the average. DPC =  dental pulp cells. PDLCs  =  periodontal ligament cells.
(XLS)

Table S3 **List of the designed oligonucleotides containing 3 tandem copies of miR-720 predicted target site in NANOG 3′-UTR.** Mutant 1 and Mutant 2 correspond to point mutations in the seed sequence of miR-720 predicted target site in NANOG 3′-UTR.
(XLSX)
