# Supplementary figures and images for: miRNA-720 Controls Stem Cell Phenotype, Proliferation and Differentiation of Human Dental Pulp Cells
Source: PLoS One. 2013 Dec 30;8(12):e83545. doi: 10.1371/journal.pone.0083545 (PMC3875457; doi:10.1371/journal.pone.0083545)

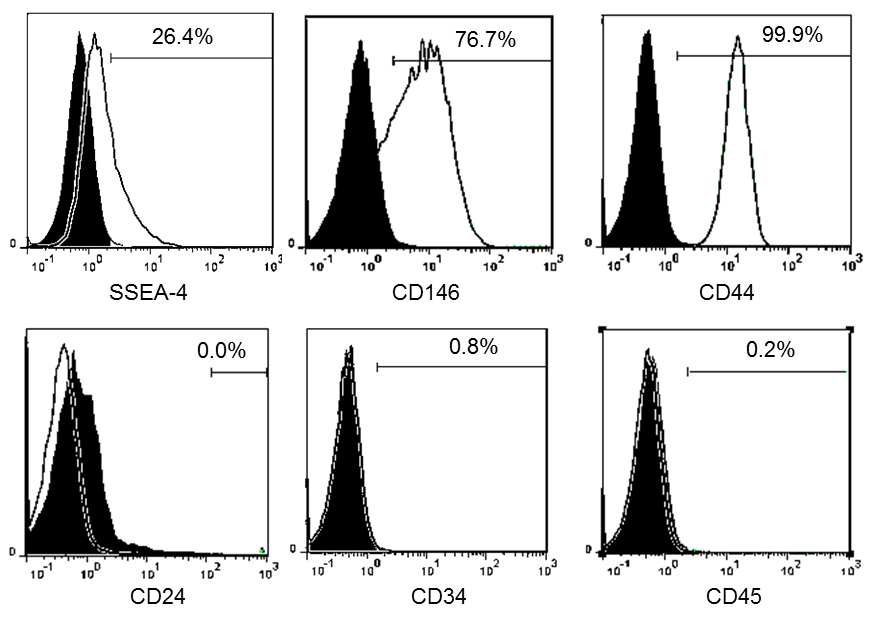

Supplement: Figure S1 — Characterization of DPCs by stem cell-related surface markers by FCM analysis. DPCs were positive to SSEA-4, CD146 and CD44; and negative to CD24, CD34 and CD45. (TIF) [file pone.0083545.s001.tif]
